# Targeting leukemia stem cells: *in vitro* veritas?

**DOI:** 10.18632/oncotarget.1777

**Published:** 2014-02-14

**Authors:** Heather M. Bond, Maria Mesuraca, Giovanni Morrone

**Affiliations:** Dept. of Experimental and Clinical Medicine - Laboratory of Molecular Haematopoiesis and Stem Cell Biology - University Magna Graecia, Catanzaro, Italy

In the past two decades it has become increasingly recognized that, in several types of cancer, only a fraction of the neoplastic cells is capable of propagating tumors upon transplantation whereas the majority of tumor cells lacks this capacity. This is particularly true for myeloid leukemias, where a minute subset of the leukemic cell population – termed leukemia stem cells (LSCs) or leukemia-initiating cells (L-ICs) - is believed to drive leukemia development and, when these cells escape chemotherapy, to cause the occurrence of relapses.

The therapeutic challenges posed by this model of leukemogenesis have spurred the active quest for novel strategies for the eradication of the LSC compartment. Diverse approaches have been proposed, aimed at targeting LSCs through their surface-membrane molecules, interfering with their cell-cycle regulation, signalling pathways, DNA damage response, metabolic properties, genetic or epigenetic features, interactions with the microenvironment [1]. Such strategies have indeed led to the discovery of several candidate therapeutic agents [1-3], some of which are currently being tested in clinical trials.

Systematic high-throughput screenings of collections of small molecules with therapeutic potential hold promise to yield novel effective drugs to target L-ICs, but have rarely been pursued thus far. One main limitation for this approach is the difficulty of obtaining, and propagating in culture, adequate amounts of L-ICs. This obstacle may be circumvented using experimental models of myeloid leukemogenesis based on the retro- or lentiviral transduction of normal hematopoietic stem and progenitor cells (HSPCs) with leukemia-associated oncogenes. The enforced expression of these oncogenes, alone or in combination, confers on the transduced cells features similar to those of L-ICs (including extended self-renewal and limited differentiation potential), thereby generating transformed cell lines enriched in leukemia stem-like cells.

**Figure 1:**
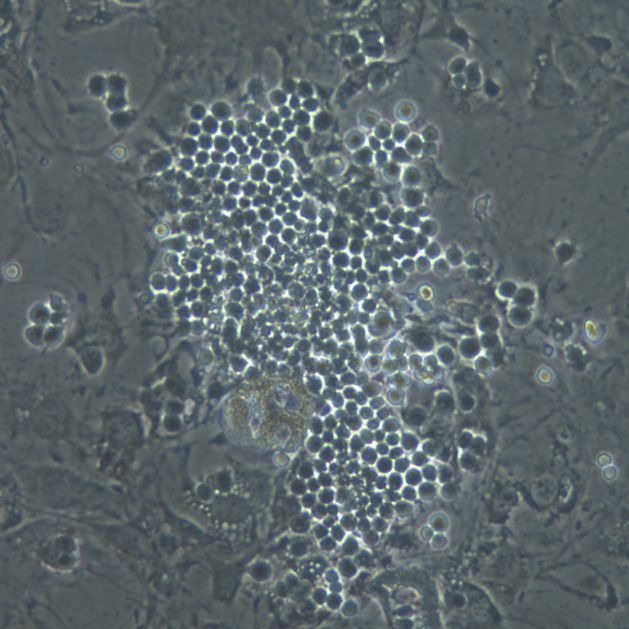
Cobblestone area Phase-contrast picture of a co-culture of human cord blood-derived CD34+ cells with the mouse stromal cell line, MS-5. In addition to the cluster of small round cells constituting the cobblestone and to the stromal cells (some of which undergoing adipocytic differentiation), refrangent hematopoietic cells in suspension can be observed

A recently-published report [4] illustrates an extensive screening carried out in the framework of a multi-institutional collaboration among the laboratories of Malcolm Moore, David Scadden, Stuart Schreiber, Benjamin Ebert and Todd Golub. This team devised a sophisticated strategy to assess the effects of almost 15,000 synthetic small molecules on the most primitive leukemic cells within the context of the bone marrow microenvironment. Murine myeloid progenitors expressing the fluorescent protein dsRed, transduced with the MLL-AF9 oncogene, were serially transplanted in irradiated hosts where they generated leukemias with increasingly short latency; LSCs were isolated from the bone marrow of quaternary recipients and co-cultured with stromal cells expressing GFP. To identify compounds selectively inhibiting LSCs but not normal HSPCs, the authors used as a readout the formation of cobblestone areas (CAs). These are clusters of small, round and phase-dark hematopoietic cells embedded in the stromal layer, derived from immature progenitors (cobblestone area-forming cells, CAFCs) that migrate and settle beneath the stroma and - after a variable latency that depends on their immaturity - begin to proliferate and generate structures that resemble cobblestones (Fig. 1). In addition to normal HSPCs, also leukemic or oncogene-transformed early progenitors can form cobblestone areas [5-6], and this property was exploited by Hartwell et al. to identify compounds with inhibitory activity on leukemic, but not normal CAFCs. As scoring CAs is extremely laborious, an automated image analysis system, trained for CA recognition, was developed to enumerate the dsRed-positive CAs in the GFP-expressing stromal monolayers. Through multiple screenings, 155 compounds were found to effectively inhibit leukemic, but not normal CAFCs, several of which with an EC_50_ in the low sub-micromolar range, thereby providing a pool of potentially effective anti-L-IC agents for future studies. Some of these were already known to target LSCs, like the sesquiterpene lactone, parthenolide [1]; some compounds acted exclusively on CAFCs, others exerted their inhibitory effects both through cell-intrinsic and extrinsic (stroma-mediated) mechanisms. One of the most potent and selective compounds identified was lovastatin, that was further assayed on six primary LSC-enriched human AML samples harboring different genetic aberrations. Lovastatin inhibited CAFC formation in all these samples, with an EC_50_ (<250nM) similar to that observed with mouse LSCs. Other statins also displayed LSC-inhibitory activity, that appeared to depend strictly on the inhibition of the HMG-CoA reductase. *Ex-vivo* pretreatment with lovastatin of co-cultured LSCs and normal HSPCs effectively prevented leukemia development, but not hematopoietic reconstitution when the cells were co-transplanted in irradiated host.

Thus, inhibitors of mevalonate synthesis may represent new weapons against chemotherapy-resistant LSCs, although their mechanism of action remains to be fully clarified and new formulations should be studied to ensure effective concentrations in plasma and bone marrow. The panel of new molecules identified in this study may be further expanded using other models of AMLs driven by different oncogenes (or combinations thereof) that are already available: one could envision that in the near future extensive arrays of validated compounds may become available, to assess the sensitivity of individual relapsed or refractory AMLs. But beyond its finding, albeit translationally relevant, the study of Hartwell et al. demonstrates that a complex *in vitro* system, that faithfully mimics the interactions occurring *in vivo* between malignant stem cells and stromal microenvironment, can be successfully exploited for the high-throughput discovery of novel antineoplastic agents to target the elusive cancer- (and/or metastasis-) initiating cells compartment.
